# Mechanism of Cancer Cell Death Induced by Depletion of an Essential Replication Regulator

**DOI:** 10.1371/journal.pone.0036372

**Published:** 2012-05-04

**Authors:** Sayuri Ito, Ai Ishii, Naoko Kakusho, Chika Taniyama, Satoshi Yamazaki, Rino Fukatsu, Asako Sakaue-Sawano, Atsushi Miyawaki, Hisao Masai

**Affiliations:** 1 Genome Dynamics Project, Department of Genome Medicine, Tokyo Metropolitan Institute of Medical Science, Setagaya-ku, Tokyo, Japan; 2 Laboratory for Cell Function and Dynamics, Advanced Technology Development Group, Brain Science Institute, RIKEN, Wako-city, Saitama, Japan; Rush University Medical Center, United States of America

## Abstract

**Background:**

Depletion of replication factors often causes cell death in cancer cells. Depletion of Cdc7, a kinase essential for initiation of DNA replication, induces cancer cell death regardless of its p53 status, but the precise pathways of cell death induction have not been characterized.

**Methodology/Principal Findings:**

We have used the recently-developed cell cycle indicator, Fucci, to precisely characterize the cell death process induced by Cdc7 depletion. We have also generated and utilized similar fluorescent cell cycle indicators using fusion with other cell cycle regulators to analyze modes of cell death in live cells in both p53-positive and -negative backgrounds. We show that distinct cell-cycle responses are induced in p53-positive and -negative cells by Cdc7 depletion. p53-negative cells predominantly arrest temporally in G2-phase, accumulating CyclinB1 and other mitotic regulators. Prolonged arrest at G2-phase and abrupt entry into aberrant M-phase in the presence of accumulated CyclinB1 are followed by cell death at the post-mitotic state. Abrogation of cytoplasmic CyclinB1 accumulation partially decreases cell death. The ATR-MK2 pathway is responsible for sequestration of CyclinB1 with 14-3-3σ protein. In contrast, p53-positive cancer cells do not accumulate CyclinB1, but appear to die mostly through entry into aberrant S-phase after Cdc7 depletion. The combination of Cdc7 inhibition with known anti-cancer agents significantly stimulates cell death effects in cancer cells in a genotype-dependent manner, providing a strategic basis for future combination therapies.

**Conclusions:**

Our results show that the use of Fucci, and similar fluorescent cell cycle indicators, offers a convenient assay system with which to identify cell cycle events associated with cancer cell death. They also indicate genotype-specific cell death modes induced by deficient initiation of DNA replication in cancer cells and its potential exploitation for development of efficient cancer therapies.

## Introduction

Cdc7 is a conserved serine-threonine kinase which plays a critical role in the firing of replication origins [1]–[3]. A key substrate is MCM, a component of the prereplicative complex (pre-RC), and phosphorylation of the MCM2, 4 and 6 subunits of the MCM complex by Cdc7 triggers the association of Cdc45 with pre-RC, a crucial step for generation of an active replication fork [4]–[6]. Cdc7 forms a complex with Dbf4, an activation subunit, to generate an active kinase complex [2]. In humans, two activation subunits, ASK and Drf1/ASKL1, are known to exist [2], [7]–[9].

Knockout of Cdc7 in mice causes early embryonic lethality. Inactivation of Cdc7 genes in mouse ES cells is also lethal [10]; cells cease DNA synthesis, accumulate DNA damages, and eventually undergo cell death in a p53-dependent manner. Knockdown experiments in mammalian cells indicate that ASK is essential while Drf1/ASKL1 may be dispensable for viability [9], [11]. Indeed, inactivation of the ASK genes in mouse ES cells also leads to lethality [12]. These results indicate that Cdc7-ASK is essential for proliferation of mammalian cells. On the other hand, Drf1/ASKL1 may play a predominant role as an activator of Cdc7 in the early development of amphibians [13], [14]. An ortholog of Drf1/ASKL1 has not been identified in mice.

On a cellular level, knockdown of Cdc7 was shown to cause cell death in cancer cells, but not in normal cells, in which p53-dependent pathways arrest the cell cycle presumably in G1 phase [15], [16]. It was also reported that Cdc7 knockdown induced p38-dependent cell death in HeLa cells [17]. However, Cdc7 depletion causes cell death also in p53-positive cells, suggesting that p53 alone cannot prevent cell death induced by Cdc7 depletion in cancer cells. At present, the precise mechanisms of p53-independent cell death in Cdc7-depleted cancer cells are not known. In this study, we analyzed the effect of Cdc7 depletion in cancer cells by using the recently developed cell cycle indicator Fucci [18] as well as similar fluorescent cell cycle indicators. Our results point to differential effects of p53 on the mode of cell death in Cdc7-depleted cancer cells.

## Results

### Depletion of Cdc7 kinase in human cancer cells causes cell death

Depletion of Cdc7 in HeLa, U2OS or other cancer cells with siRNA resulted in inhibition of DNA synthesis, accumulation of chromosome damages [represented by γ-H2AX foci) and eventual loss of viability viability [15], [19], [20]. Cell death was induced in both p53-positive or p53-negative cancer cells, consistent with previous reports [15], [19]. FACS analyses of DNA content indicated that Cdc7 depletion leads initially to decreased G1 population, followed by increase of sub-G1 population, indicative of cell death (**Fig. S1A and Fig. S2B**).

**Figure 1:**
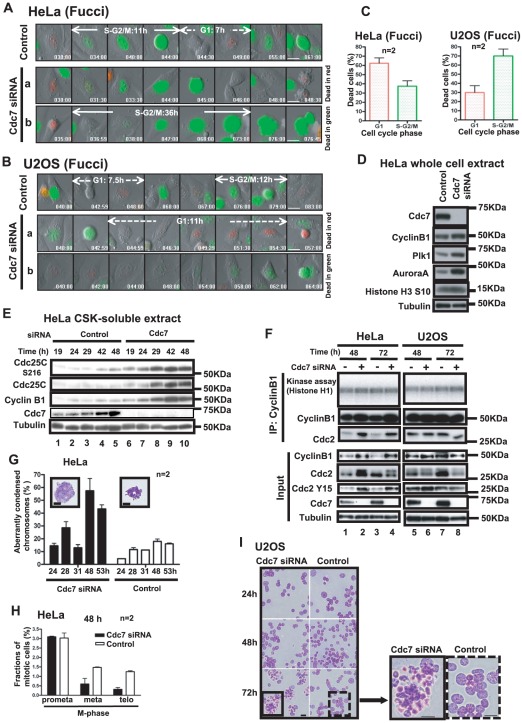
Cdc7 depletion in cancer cells induces cell death: effect on Cdc2-CyclinB1 and mitosis. (**A and B**) HeLa (**A**) or U2OS (**B**) cells expressing Fucci were treated with control or Cdc7-D siRNA, and time lapse image was recorded with Olympus LCV100 (**movies S1 and S2**). Images taken from the time lapse data at the times indicated are presented. The uppermost panels (control siRNA) indicate cells undergoing normal cell division. Numbers in each panel show time (hrs) after siRNA transfection. Lower two panels (**a** and **b**) show Cdc7 siRNA treated cells. Some cells died in red color (G1 phase, **a**), and other cells died in green (S/G2/M phase, **b**). Lengths of cell cycle stages are indicated in the panels (G1, arrowed broken lines; S/G2/M, arrowed solid lines). Bar, 20 µm. (**C**) Dead cells in Cdc7 siRNA-treated HeLa-Fucci (left, 324 cells) or U2OS-Fucci (right, 180 cells) were counted from the time lapse data to determine the fractions of the dead cells in red and in green. Cell death occurs at both G1 and S/G2/M phases in Cdc7 siRNA treated cancer cells. (**D, E and F**) HeLa cells were transfected with control or Cdc7-D siRNA and were harvested at 48 hrs (**D**) or at the times indicated (**E and F**). The whole cell extracts (**D**) or CSK-soluble extracts (**E**) were analyzed by western blotting using the antibodies indicated. (**F**) Cdc2-CyclinB1 kinase activity was measured using the CSK-soluble extracts. The immunoprecipitates (IP) used for the assays and the input extracts were analyzed by western blotting. The extent of Cdc7 depletion was similar between HeLa and U2OS. Cdc7 was not detectable by western after siRNA treatment in both cells. (**G**) HeLa cells were treated with control or Cdc7-D siRNA for indicated times, collected, washed with PBS, swollen in 75 mM KCl for 20 min at 37°C, and fixed with glacial acetic acid/methanol (1∶3) solution three times. Fixed chromosomes were dropped on a slide glass, air dried and stained with 5% Giemsa's solution in 1/15 M PBS. Spread chromosomes were observed under All-in-One microscopy (Keyence). The mitotic cells with aberrantly condensed chromosomes were counted and the fractions are presented. The insets show representative images of aberrantly condensed chromosomes observed in a Cdc7 siRNA treated HeLa cell (left) and properly condensed chromosomes observed in a control cell (right). Bar, 50 µm. (**H**) HeLa cells were treated with control or Cdc7-D siRNA for 48 hrs, washed with PBS, fixed with 4% paraformaldehyde for 10 min at room temperature and then stained with Hoechst 33342. Cells were examined under confocal microscopy LSM510 (1427 cells [Cdc7] and 1023 cells [control]), and the cells in M phase stages were scored. The fractions of cells in each mitotic stage are presented. (**I**) Spread and fixed chromosomes prepared in U2OS as described above were observed by FSX100 Olympus microscopy. No significant difference was observed in mitotic cells after Cdc7 depletion. However, the numbers of apoptotic cells increased in Cdc7-depleted U2OS cells. Bar, 32 µm. In C, G and H, “n” represents the numbers of independent experiments conducted.

In order to investigate the mode of cell death induced by Cdc7 depletion, we used HeLa cells expressing the cell cycle indicator, Fucci (Fluorescent ubiquitin-based cell cycle indicator; [18]), which permits visualization of the cell cycle state (red for G1 and green for S/G2/M). HeLa-Fucci was transfected with Cdc7 siRNA and the cells were monitored to determine the cell cycle stage at which they undergo cell death. Cell death occurs at both post-mitotic G1 and during S/G2/M phase in HeLa-Fucci (**Fig. 1A and C** **, movies S1 and S2**). We also generated U2OS-Fucci and examined the cell death mode in U2OS after Cdc7 depletion. In U2OS, more than 70% of the cells died during S/G2/M phase (green; **Fig. 1B and C**). In contrast, Cdc7 depletion did not induce cell death in NHDF (normal human dermal fibroblast) cells and led mostly to G1 arrest as described previously (**Fig. S1 and data not shown;** [15]).

### Cytoplasmic accumulation of CyclinB1 protein in Cdc7-depleted HeLa cells

The FACS analysis of Cdc7-depleted HeLa cells did not show accumulation of G2/M population (**Fig. S1A and Fig. S2B**). However, Western analyses of various proteins after Cdc7 depletion in HeLa cells indicated that levels of CyclinB1, AuroraA and Plk1 proteins increased (**Fig. 1D, E and F**). The levels of both Cdc25C, Cdc25CS216 (**Fig. 1E**) and the tyrosine 15 phosphorylation of Cdc2 also increased (**Fig. 1F**), suggesting that G2/M checkpoint may be induced in Cdc7-depleted HeLa cells. Although the level of CyclinB1 protein increased, the CyclinB1-dependent Cdc2 kinase activity was almost the same as that of the control (**Fig. 1F**). This may be due to the association with 14-3-3 proteins, which may inhibit the kinase activity (see below), as well as to the checkpoint-induced inhibitory tyrosine 15 phosphorylation. On the other hand, in p53-positive U2OS, depletion of Cdc7 did not cause CyclinB1 accumulation, and did not affect CyclinB1-dependent Cdc2 kinase activity or tyrosine 15 phosphorylation of Cdc2 (**Fig. 1F**).

The staining and observation of M phase chromosomes indicated an increase of aberrantly condensed chromosomes in Cdc7 siRNA-treated cells (**Fig. 1G**). About 50% of the mitotic cells exhibited the aberrantly condensed chromosomes in Cdc7-depleted HeLa cells. Also, metaphase to telophase cell populations decreased in Cdc7 siRNA-treated HeLa cells (**Fig. 1H**). These results indicate that a large population of cells arrest or slow down at G2 after depletion of Cdc7 kinase in HeLa cells with aberrantly condensed chromosomes, and a portion of the cells die during G2/M phase, possibly by metaphase. However, precise timing of cell death during M phase has not been determined. In U2OS, there is no significant difference for M phase chromosomes between Cdc7-depleted and control cells. However, the numbers of apoptotic nuclei increased after depletion of Cdc7 in U2OS (**Fig. 1I**). Thus, these results also show that the timing of cell death induced by Cdc7 depletion may differ in HeLa and U2OS cells.

**Figure 2:**
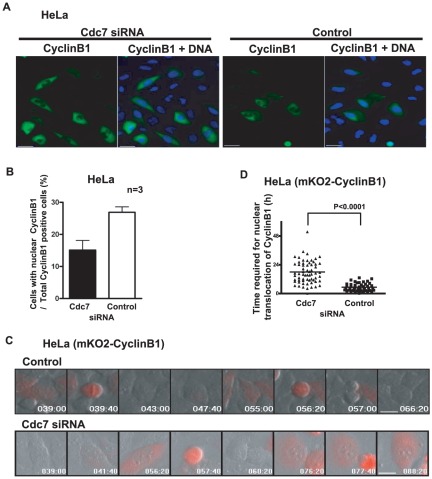
Cdc7 depletion in HeLa cells leads to accumulation of cytoplasmic CyclinB1. (**A**) HeLa cells were cultured on cover glasses, transfected with control or Cdc7-D siRNA for 48 hrs, fixed with 4% paraformaldehyde and stained by anti-CyclinB1 antibody followed by FITC-conjugated anti-mouse IgG and Hoechst33342. Left, Cdc7 siRNA; right, control siRNA. Green, CyclinB1; blue, DNA. Photos were taken by FSX100 Olympus microscopy. Bar, 16 µm. (**B**) More than 3,000 cells were examined and cells with nuclear CyclinB1 signals were scored and the fractions are presented. “n” represents the numbers of independent experiments conducted. (**C**) HeLa cells expressing mKO2-CyclinB1 were treated with Cdc7-D siRNA or control siRNA. Time lapse images were recorded by Olympus LCV100 (**movies S3 and S4**). Images taken from the time lapse data at the times indicated are presented. Upper, control siRNA; lower, Cdc7 siRNA. Red signals show mKO2-CyclinB1. The control siRNA-treated cells indicate those undergoing periodic cytoplasmic appearance, nuclear transfer, and degradation (upper panel), whereas the Cdc7 siRNA-treated cells show persistent strong cytoplamic signals for a long period (lower panel). Numbers in each panel show time (hrs) after siRNA treatment. Bar: 20 µm. (**D**) The time (hr) between the first appearance of cytosolic mKO2-CyclinB1 signal and its translocation into the nucleus was measured in the time lapse images of control or Cdc7-D siRNA treated HeLa cells. The P-value of the two-tailed unpaired t-test was calculated by Prism software.

We then analyzed the cellular localization of CyclinB1 protein by immunostaining. We noted the increase of CyclinB1-positive cells in Cdc7 siRNA-treated cells, as expected from an increase of the overall CyclinB1 protein level. We also noted that a substantial population of Cdc7-depleted HeLa cells contain CyclinB1 protein in cytoplasm (**Fig. 2A and B**). To confirm this result, we examined the effect of Cdc7 depletion using HeLa cells stably expressing mKO2-fused CyclinB1 protein. In this cell line, the red fluorescent signals first appeared in cytoplasm at about 10–14 hrs after cell division. The signals were detectable for about 5–6 hrs. Since the synchronization experiments suggest that G2/M phases in HeLa cells last for about 3–5 hrs, CyclinB1 is likely to be expressed from late S phase through metaphase. These signals translocate into nuclei and disappear after metaphase (**Fig. 2C** **, upper panel; movie S3**), consistent with the expected behavior of the endogenous CyclinB1 protein, as shown previously [21]–[23]. When cells were treated with Cdc7 siRNA, the population of the cells with strong cytoplasmic red signals increased, and these signals stayed in the cytoplasm for a longer period (**Fig. 2C** **, lower panel and movie S4**). The time required for translocation of the red signals into nuclei after its appearance in the cytoplasm was a few hrs in the control cells, whereas it increased in Cdc7-depleted cells (**Fig. 2C and D** **, movies S3 and S4**). This was also observed with different Cdc7 siRNAs (**Fig. S2 and data not shown**). These results are consistent with the idea that CyclinB1 accumulates in the cytoplasm in HeLa cells treated with Cdc7 siRNA. We also generated HeLa cells expressing mKO2-AuroraA. Expression and activity of AuroraA, one of the mitotic kinases, is known to peak at the G2/M phase [24]. Consistently, the AuroraA signals appeared at G2 phase, and disappeared at the end of M phase in control cells, while the duration of the AuroraA signals became much longer after Cdc7 depletion (**Fig. S3, movies S5 and S6**). This effect was again seen with other Cdc7 siRNAs (**Fig. S3C and data not shown**). These results indicate that Cdc7 depletion causes the G2 cell cycle delay in HeLa cells concomitant with increased CyclinB1 and AuroraA protein levels.

Many Cdc7-depleted cells with high cytoplasmic CyclinB1 abruptly enter mitosis after lengthy G2 arrest, and very often undergo apparent cell death in the following hours. This is similar to the mitotic catastrophe reported previously [25], but the cells are restrained from proceeding into M phase by inhibition of nuclear translocation of CyclinB1, not at the stage of spindle checkpoint, as reported previously in a different system [26]. Indeed, abrogation of the spindle checkpoint by siRNA targeted to Mad2 did not affect the CyclinB1 retention in cytoplasm that occurs in response to Cdc7 depletion in HeLa cells (data not shown).

### 14-3-3σ sequesters CyclinB1 in the cytoplasm after Cdc7 depletion

**Figure 3:**
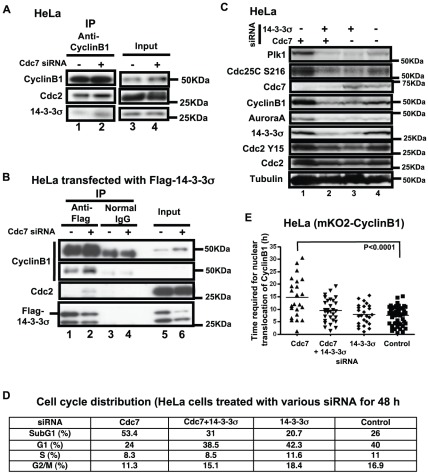
14-3-3σ sequesters CyclinB1 in the cytoplasm in Cdc7-depleted HeLa cells. (**A**) HeLa cells were treated with control or Cdc7-D siRNA for 24 hrs. Extracts were prepared and the immunoprecipitates with anti-CyclinB1 antibody (lanes 1 and 2) and input extracts (lanes 3 and 4; 20% of the extract used for immunoprecipitation) were analyzed by western blotting. (**B**) HeLa cells were treated with control or Cdc7-D siRNA, followed by transfection of a Flag-tagged 14-3-3σ-expressing plasmid. Extracts were prepared at 48 hrs after siRNA transfection and the immunoprecipitates with anti-Flag antibody (lanes 1 and 2) or normal (control) IgG (lanes 3 and 4) and input extracts (lanes 5 and 6; 17% of the extract used for immunoprecipitation) were analyzed by western blotting. The binding of Cdc2/CyclinB1 with 14-3-3σ increases after Cdc7 siRNA treatment, suggesting that 14-3-3σ retains CyclinB1 in the cytoplasm after Cdc7 depletion in HeLa cells. (**C** and **D**) HeLa cells were treated with Cdc7-D siRNA, 14-3-3σ and Cdc7-D siRNAs, 14-3-3σ siRNA and control siRNA for 48 hrs. (**C**) Western blot analysis of the whole cell extracts. (**D**) DNA contents of the cells in **C** were analyzed by FACS (10,000 cells for each) and the fraction of the cells in each cell cycle stage is presented. (**E**) HeLa cells expressing mKO2-CyclinB1 were treated with Cdc7-D and/or 14-3-3σ siRNA as indicated. The time (hr) between the first appearance of cytosolic mKO2-CyclinB1 signals and its translocation into the nucleus was measured using the time lapse images, which started at 24 hrs after siRNA transfection. The P-value of the two-tailed unpaired t-test was calculated by Prism software. Co-depletion of 14-3-3σ led to a decrease in the overall CyclinB1 protein level (**C**), reduced cell death (**D**), and reduced the duration of its cytoplasmic retention (**E**).

The next question is how CyclinB1 accumulates in the cytoplasm. 14-3-3σ is conserved, well-characterized factors, known to bind to various cell cycle regulators and retain them in cytoplasm in some circumstances [25]. Each of the seven 14-3-3 isoforms was expressed, and its interaction with Cdc2-CyclinB1 was examined. 14-3-3σ was among the strongest binders (data not shown). We examined whether the accumulated CyclinB1 is bound to 14-3-3σ in Cdc7-depleted HeLa cells and found that CyclinB1-bound 14-3-3σ significantly increased in Cdc7-depleted cells (**Fig. 3A** **, lane 2**). Also, immunoprecipitation of transiently expressed 14-3-3σ after Cdc7 depletion showed that CyclinB1 and Cdc2 are associated with 14-3-3σ (**Fig. 3B**). However, we failed to detect the association of 14-3-3σ and Cdc25C, as previously described [25], [27]. These results suggest that 14-3-3σ sequesters the Cdc2-CyclinB1 complex in the cytoplasm after Cdc7 depletion in HeLa cells.

### Reduction of cytoplasmic accumulation of CyclinB1 partially reduces cell death

Since cells accumulating CyclinB1 in the cytoplasm are prone to cell death, we examined if reduction of cytoplasmic CyclinB1 antagonizes the cell death effect of Cdc7 depletion. Co-depletion of both Cdc7 and 14-3-3σ in HeLa cells reduced the CyclinB1, AuroraA, Plk1 and Cdc25C protein levels (**Fig. 3C**). The time required for nuclear translocation of CyclinB1 shortened and the sub-G1 cell population decreased (**Fig. 3D and E**). These findings suggested that the absence of 14-3-3σ facilitates the G2-M transition and progression into the next cell cycle stage, partially rescuing the cells from cell death.

**Figure 4:**
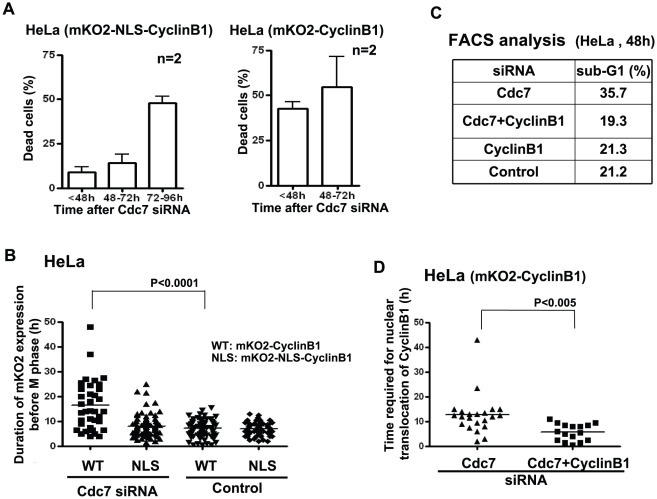
Expression of nuclear localization signal-targeted CyclinB1 partially reduces cell death in Cdc7-depleted HeLa cells. (**A**) HeLa cells expressing mKO2-NLS-CyclinB1 (left) or mKO2-CyclinB1 (right) were treated with control or Cdc7-D siRNA and were monitored by Olympus LCV100. Dead cells were counted in the time lapse images at the times indicated. Cell death was suppressed in mKO2-NLS-CyclinB1 expressing HeLa cells up to 72 hrs (at which half of the Cdc7-depleted HeLa cells are usually dead). mKO2-NLS-CyclinB1 plasmid was constructed by inserting the NLS sequence (PPKKKRKVEDP) from the SV40 large T antigen into the mKO2-CyclinB1 plasmid between mKO2 and CyclinB1. About 200 or 60 cells expressing mKO2-NLS-CyclinB1 or mKO2-CyclinB1, respectively, were counted. “n” represents the numbers of independent experiments conducted. (**B**) HeLa cells expressing mKO2-CyclinB1 (WT) or mKO2-NLS-CyclinB1 (NLS) were treated with control or Cdc7-D siRNA and the duration of CyclinB1 expression before entry into M phase was measured in the time lapse images. Upon Cdc7 depletion, NLS cells did not accumulate the tagged CyclinB1 in cytoplasm, and continued through the cell cycle more or less normally. (**C**) HeLa cells were treated with indicated siRNAs for 48 hrs. DNA contents were analyzed by FACS (10,000 cells for each) and the fractions of sub-G1 population were calculated and presented. Co-depletion of CyclinB1 reduced the cell death induced by Cdc7-D siRNA in HeLa cells. (**D**) HeLa cells expressing mKO2-CyclinB1 (WT) were treated with Cdc7-D or Cdc7-D+CyclinB1 siRNA and the time (hr) between the first appearance of cytosolic mKO2-CyclinB1 signal and its translocation into the nucleus was measured in the time lapse images of each cell population. Down-regulation of CyclinB1 expression shortened the G2 arrest induced by Cdc7 depletion. In (B) and (D), the P-values of the two-tailed unpaired t-test were calculated by Prism software.

We next expressed mKO2-NLS-CyclinB1, which constitutively localizes in nuclei in HeLa cells at late S through metaphase. In these cells, the time required for transition from late S to G2/M was shortened compared to mKO2-CyclinB1 expressing cells and cell death was partially circumvented at 48 hrs after Cdc7 depletion (**Fig. 4A and B**). This is consistent with a previous report that ectopic expression of NLS-CyclinB1 in HeLa cells reduced the G2-delay occurring in response to X-ray irradiation [28]. However, cells expressing mKO2-NLS-CyclinB1 still underwent apoptosis at later time points after Cdc7 depletion. This may be expected since expression of nuclear CyclinB1 is known to induce apoptosis in cancer cells [29]. Co-depletion of CyclinB1 reduced G2-elongation and partially rescued the cell death induced by Cdc7 depletion (**Fig. 4C, D**). These results support the notion that cytoplasmic accumulation of CyclinB1 and its abrupt translocation into nuclei are at least partially responsible for the observed cell death in HeLa cells induced by Cdc7 depletion and that the cell death can be at least partially prevented by facilitating mitosis.

### G2 elongation is caused by depletion of Cdc7 in p53-negative cells through MK2 activation

**Figure 5:**
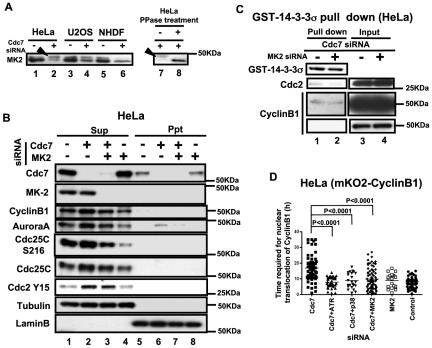
MK2 is activated in Cdc7-depleted HeLa cells and is required for cytoplasmic accumulation of CyclinB1. (**A**) HeLa (lanes 1, 2, 7 and 8), U2OS (lanes 3 and 4) and NHDF (lanes 5 and 6) cells were treated with control or Cdc7 siRNA and the whole cell extracts were run on a phosgel and analyzed by western blotting. Lanes 7 and 8, extracts from Cdc7 siRNA-treated HeLa cells were non-treated (−) or treated with λ-phosphatase (+). Arrowheads indicate the phosphorylated MK2 band. (**B**) HeLa cells were treated with control siRNA (lanes 1 and 5), Cdc7 siRNA (lanes 2 and 6), Cdc7 and MK2 siRNAs (lanes 3 and 7) and MK2 siRNA (lanes 4 and 8) for 48 hrs, and CSK-soluble (lanes 1–4; Sup) and -insoluble (lanes 5–8; Ppt) proteins were analyzed by western blotting. Tubulin and LaminB are shown as loading controls. (**C**) Glutathion Sepharose 4B beads carrying GST-14-3-3σ protein was incubated for 1 hr at 4°C with CSK-soluble extracts of HeLa cells treated with siRNA, as shown. Bound proteins were examined by Western blotting. “Input” represents only the extracts without added GST-14-3-3σ protein. Cdc7 and MK2 co-depletion reduced the binding between 14-3-3σ and Cdc2/CyclinB1. (**D**) HeLa cells expressing mKO2-CyclinB1 were treated with indicated siRNAs. The time (hr) between the first appearance of cytosolic mKO2-CyclinB1 signal and its translocation into the nucleus was measured in the time lapse images. Co-depletion of MK2, p38 (upstream kinase of MK2) or ATR reduced cytoplasmic retention of CyclinB1 (G2 elongation) was observed in Cdc7-depleted HeLa cells. The P-values of the two-tailed unpaired t-test were calculated by Prism software. Cdc7-D siRNA was used in all the experiments.

It was previously reported that the p38-MK2 pathway is activated in HeLa cells by depletion of Cdc7 [17]. Therefore, we examined whether this pathway is involved in cytoplasmic accumulation of CyclinB1 in Cdc7-depleted HeLa cells. First, we confirmed that MK2 is hyperphosphorylated by Cdc7 depletion in HeLa cells, but not in U2OS or NHDF cells (**Fig. 5A**). This activation of MK2 in Cdc7-depleted HeLa cells could be due to direct activation of MK2 by Cdc7-induced replication stress. Alternatively, increase of G2 phase cells by Cdc7 depletion may contribute to activation of MK2, since MK2 is known to be more active in G2 and M phases. Co-depletion of Cdc7 and MK2 reduced the Cyclin B1 and AuroraA protein levels and Cdc25C Ser216 phosphorylation, suggesting that mitosis is partially restored (**Fig. 5B**). It also reduced the binding of 14-3-3σ and CyclinB1/Cdc2 (**Fig. 5C**). However, co-depletion of Cdc7 and MK2 did not prevent HeLa cell death, presumably because MK2 depletion alone induced significant cell death (data not shown).

The time required for nuclear translocation after co-depletion of Cdc7 and MK2 was shortened close to the control level. Furthermore, the G2 elongation observed after Cdc7 depletion was canceled also by co-depletion of ATR or p38 with Cdc7 (**Fig. 5D**). These results indicate that this G2 checkpoint depends on ATR-regulated MK2 activation.

### Cytoplasmic accumulation of CyclinB1 does not occur in p53-positive U2OS or HCT116 after Cdc7 depletion

**Figure 6:**
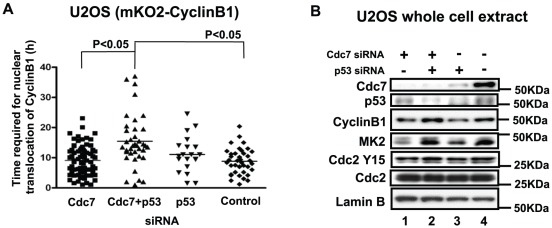
CyclinB1 does not accumulate in cytoplasm in Cdc7-depleted U2OS cells. (**A**) U2OS cells expressing mKO2-CycinB1 were treated with siRNAs and time lapse images were recorded by LCV100. The time (hr) between the first appearance of cytosolic mKO2-CyclinB1 signal and its translocation into the nucleus was measured in the time lapse images of each cell population. In Cdc7-depleted U2OS, CyclinB1 does not accumulate in cytoplasm. However, co-depletion of Cdc7 and p53 caused CyclinB1 accumulation in cytoplasm for a longer period. The P-values of the two-tailed unpaired t-test were calculated by Prism software. (**B**) Western analysis of the whole cell extracts of U2OS cells treated with indicated siRNAs for 48 hrs. A phosgel was used for the detection of MK2. Other proteins were detected on a 4–12% gradient gel.

**Figure 7:**
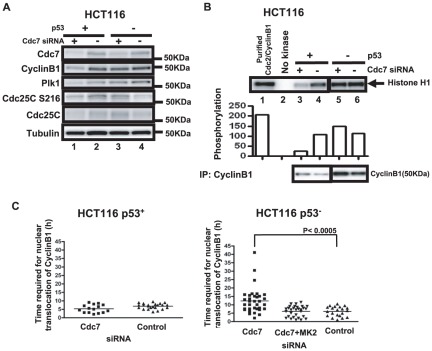
The CyclinB1 protein level and Cdc2-CyclinB1 kinase activity decrease in p53-positive HCT116 cells after Cdc7 depletion. (**A**) p53-positive or -negative HCT116 cells were treated with control or Cdc7-D siRNA for 48 hrs, and whole cell extracts were analyzed by western blotting. (**B**) CSK-soluble extracts were prepared from the same cells as in (**A**) and immunoprecipitation was conducted with anti-CylinB1 antibody. Cdc2-CyclinB1 kinase activity was measured with Histone H1 as a substrate (upper panel), as described in “Materials and Methods”. The graph below shows quantification of the level of phosphorylation. Lower panel, western blotting analyses of CyclinB1 proteins in the immunoprecipitates used for kinase assays. (**C**) p53-positive (left) or -negative (right) HCT116 cells expressing mKO2-CyclinB1 were treated with indicated siRNA and time lapse images were recorded. The time (hr) between the first appearance of cytosolic mKO2-CyclinB1 signal and its translocation into the nucleus was measured in the time lapse images. The P-values of the two-tailed unpaired t-test was calculated by Prism software.

Although Cdc7 depletion in U2OS cells (human osteosarcoma), induced vigorous cell death, the levels of the mitotic kinases did not increase (**Fig. 1**). We therefore established U2OS stably expressing mKO2-CyclinB1, and examined the effect of Cdc7 siRNA on the CyclinB1 dynamics. In this cell line, we did not observe any accumulation of CyclinB1 in the cytoplasm after Cdc7 depletion. The time required for nuclear translocation in Cdc7-depleted U2OS cells was similar to that of control cells (**Fig. 6A**). However, it became longer when p53 was co-depleted (**Fig. 6A**). The CyclinB1 protein level slightly increased after co-depletion of Cdc7 and p53 in U2OS (**Fig. 6B**). We next examined a colon cancer cell line, HCT116. In p53-positive HCT116 cancer cells, the levels of mitotic proteins such as CyclinB1 and Plk1 decreased after Cdc7 depletion presumably due to G1 arrest (**Fig. 7A**). Concomitantly, Cdc2/CyclinB1 activity was also reduced in Cdc7-depleted p53-positive HCT116 cells (**Fig. 7B**). The time required for nuclear translocation in Cdc7-depleted p53-positive HCT116 cells was similar to that of control cells (**Fig. 7C** **, left panel**). In contrast, Cdc2/CyclinB1 activity in p53-negative HCT116 after Cdc7 depletion was almost the same as that of the control (**Fig. 7B**). Furthermore, as seen in HeLa cells, the time required for nuclear translocation in Cdc7-depleted p53-negative HCT116 cells was longer than that of the control (**Fig. 7C** **, right panel**). This G2 elongation was canceled by co-depletion of Cdc7 and MK2 in p53-negative HCT116 cells (**Fig. 7C** **, right panel**). These results indicate that G2 phase elongation after Cdc7 depletion depends on the absence of p53 protein [15], [30]–[32].

**Figure 8:**
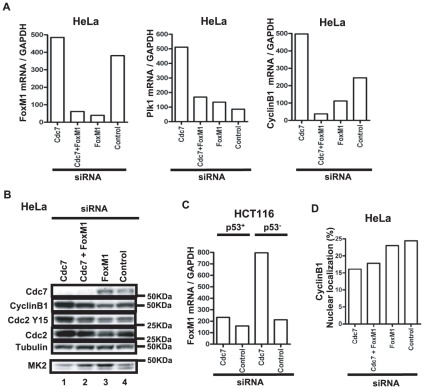
FoxM1 mRNA level increases after Cdc7 depletion in HeLa and p53-negative HCT116. (**A**) HeLa cells were treated with indicated siRNAs for 24 hrs. FoxM1 (left), Plk1 (middle) and CyclinB1 (right) mRNA levels are presented. (**B**) Western analysis of the whole cell extracts of HeLa cells treated with indicated siRNAs for 48 hrs. A phosgel was used for the detection of MK2. Other proteins were separated on a 4–12% gradient gel. (**C**) The FoxM1 mRNA levels of HCT116 (p53-positive and -negative) cells treated with control or Cdc7 siRNA for 24 hrs. In A and C, mRNA levels were quantified by real time-PCR and the relative values normalized by the level of GAPDH mRNA are presented. (**D**) HeLa cells treated with indicated siRNAs for 48 hrs were fixed with 4% paraformaldehyde for 10 min and stained with anti-CyclinB1 antibody. Fractions of the cells showing nuclear localization of CyclinB1 are shown. Cdc7-D siRNA was used in these experiments.

Recently, it was reported that the FoxO3 transcription factor is involved in G1 arrest, caused by depletion of Cdc7 in normal cells [16]. FoxM1, another Fox family member, is known to regulate expression of mitotic regulators such as Plk, CyclinB1 and CyclinA after DNA damage [33]–[36]. Expression of FoxM1 is negatively regulated by p53 [37], [38]. In HeLa, we show that the mRNA levels of FoxM1 increased after Cdc7 depletion (**Fig. 8A**), which may also be partly responsible for an increase of FoxM1 protein levels under the same condition. These findings suggest that these Fox family transcription factors may be involved in cell cycle arrest and induction of cell death by Cdc7 depletion in p53-negative cells. Double knockdown of Cdc7 and FoxM1 in HeLa cells dramatically reduced the CyclinB1 mRNA level compared to Cdc7 depletion alone. The CyclinB1 protein level also decreased by co-depletion of Cdc7 and FoxM1, but not to the extent of the mRNA level (**Fig. 8B**). FoxM1 depletion, however, did not abrogate the G2 arrest or reduce cell death (**Fig. 8D** **, data not shown**). In p53-positive HCT116 cells, the mRNA levels of FoxM1 did not change significantly, probably due to negative regulation of FoxM1 expression by p53 [37]. CyclinB1 and Plk mRNA levels also did not change after Cdc7 depletion in this cell line (**data not shown**). Thus, increased expression of FoxM1 may be at least partially responsible for the increased CyclinB1 protein level in Cdc7-depleted HeLa cells, which are deficient in p53, although it may not be essential for cytoplasmic accumulation of CyclinB1 and induced cell death. However, these results strongly suggest that cytoplasmic sequestration and accumulation of CyclinB1 is a predominant factor for cell death in p53-negative cells.

### Efficient induction of cell death in cancer cells by combination of Cdc7 siRNA and conventional anti-cancer agents

Combinational therapy is sometimes efficient in treating cancer patients. The results described above and from other reports indicate that Cdc7 could be a novel effective target for cancer therapy, the inhibition of which might induce cancer cell-specific cell death through novel and distinct pathways in both p53-positive and -negative cancer cells [15], [30]–[32]. We used p53-positive and -negative HCT116, a colon cancer cell line, and compared the effects of Cdc7 depletion. As reported previously, both cells underwent cell death after Cdc7-depletion. We then examined the effect of conventional cancer treatment genotoxic agents, etoposide (topoisomerase II inhibitor) or 5FU (5′ fluorouracil; irreversible inhibitor of thymidylate synthase), which would inhibit the DNA chain elongation process, for cell death-inducing effect of Cdc7 siRNA or a Cdc7 inhibitor in p53-positive and -negative HCT116 cells.

**Figure 9:**
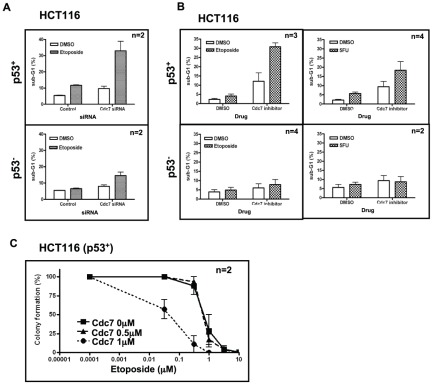
Coadministration of conventional anti-cancer drugs enhances cell death in a p53-dependent manner. (**A**) p53-positive (upper) or -negative (lower) HCT116 cells were treated with Cdc7-D or control siRNA for 24 hrs, followed by treatment with DMSO or 10 µM etoposide (Wako) for 32–40 hrs. (**B**) p53-positive (upper) or -negative (lower) HCT116 cells were treated with indicated chemicals (10 µM etoposide or 5FU (Wako) and 1 µM Cdc7 inhibitor (Calbiochem)) for 16 hrs. In A and B, DNA contents were analyzed by FACS (10,000 cells for each) and the fractions of sub-G1 population were calculated and presented. Synergistic effect of etoposide and 5FU on cell death induced by Cdc7 inhibition was observed in a p53-positive HCT116 but not in p53-negative HCT116. (**C**) Colony formation assays were conducted in p53-positive HCT116 cells. Cells treated with drugs for 16 hrs were collected and 500 cells were seeded in a 3.5 cm plate and cultured. The numbers of colonies were counted after 7 days. “n” represents the numbers of independent experiments conducted.

We noted that the co-treatment with etoposide synergistically increased the sub-G1 population in Cdc7 siRNA-treated p53-positive HCT116 compared to the cells treated with the drug alone. This stimulation of cell death by co-treatment of the Cdc7 depletion and the genotoxic agents was not observed in p53-negative HCT116. (**Fig. 9A**). Furthermore, we noted that the co-treatment with etoposide or 5FU with a Cdc7 chemical inhibitor also significantly increased the sub-G1 population in p53-positive, but not in p53-negative HCT116 cells (**Fig. 9B**). We next conducted colony formation assays after treating the cells with a combination of etoposide and the Cdc7 chemical inhibitor. The synergistic effect of etoposide and the Cdc7 inhibitor on colony formation was once again observed in p53-positive HCT116 cells only (**Fig. 9C**). These results suggest a potential novel cancer therapy involving the combination of Cdc7 inhibition and conventional anti-cancer agents that is effective specifically in p53-positive cancer cells.

## Discussion

### Cancer cell death induced by Cdc7 depletion

The blocking of DNA replication in eukaryotic cells normally induces checkpoint responses which slow down the ongoing forks and prevent firing of new replication origins. This would sustain cell viability by restraining aberrant DNA replication. In cancer cells, the cells often enter lethal S phase due to partial defect in checkpoint pathways. Indeed, normal cells would undergo cell death by arrest of DNA replication forks when one or more of the checkpoint pathways are shut down [15], [16], [39].

In many cancer cells, Cdc7 depletion causes vigorous cell death, which is independent of the p53 status. Use of Fucci, a fluorescent cell cycle indicator, as well as other fluorescent marker genes, permitted us to determine the cell cycle stage at which cells undergo cell death upon depletion of Cdc7. Cdc7-depleted HeLa-Fucci cells die at both red (G1) and green (S/G2/M) states. On the other hand, about 70% of the cell death in Cdc7-depleted U2OS-Fucci cells appears to occur in “green” cells and predominantly during S phase. This conclusion is consistent with our observation that U2OS cells expressing mKO2-CyclinB1 rarely underwent cell death when mKO2 was expressed (indicative of late S/G2/M). Thus, timing of cell death induced by Cdc7 depletion may differ in HeLa and U2OS cells. These results indicate that Cdc7 depletion-dependent cell death can occur through multiple mechanisms and that the p53 state may affect the choice of cell death modes.

### G2 cell cycle elongation induced by Cdc7 depletion is dependent on the p53 status

We showed that CyclinB1 accumulates in the cytoplasm of Cdc7-depleted HeLa cells, but not in p53-positive U2OS or HCT116 cells. The accumulation of CyclinB1 is due to 14-3-3σ, and co-depletion of 14-3-3σ leads to abrogation of CyclinB1 accumulation as well as partial rescue of viability. ATR-MK2, activated by Cdc7 depletion, is required for CyclinB1 accumulation. Abrogation of CyclinB1 accumulation by other methods also resulted in less cell death, indicating that the cytoplasmic sequestration/accumulation of CyclinB1 and the following abrupt transport into nuclei may be a predominant factor for cell death in p53-negative cells.

It was reported in hematopoietic cells that ectopic overexpression of CyclinB1 causes apoptosis. Furthermore, the elevated level of CyclinB1 stimulates γ-irradiation induced cell death [40]. It was also reported that nuclear accumulation of CyclinB1 causes apoptosis in cancer cells [29]. We see more than half of the cell population die after translocation of the accumulated CyclinB1 into nuclei, which causes transient but marked increases of nuclear CyclinB1, leading to aberrant chromosome separation and cell division. We also observed cell death in those cells accumulating CyclinB1 in cytoplasm (**Fig. 2C**).

In p53-positive cells, in contrast, Cdc7 depletion led predominantly to death during S phase. This may be due to p53-mediated G1 or S phase arrest, that eventually leads to aberrant entry into S phase. FoxM1 is required for transcriptional up-regulation of mitotic regulators in Cdc7-depleted HeLa cells (**Fig. 8**). p53-mediated inhibition of FoxM1 may also contribute to reduced mitotic regulators in Cdc7-depleted p53-positive cancer cells.

### Addition of conventional anti-cancer drugs facilitates Cdc7 depletion-induced cancer cell death

Combinations of multiple anti-cancer drugs can sometimes be more effective and have less side effects when treating cancer patients than the use of single anti-cancer drugs. However, the rationale behind effective multi-drug cancer therapy strategies has not been well established. We examined the effect of etoposide and 5FU, frequently-used anti-cancer agents, on Cdc7 inhibition-induced cell death in p53-positive or p53-negative HCT116 cells. The Cdc7 inhibitor used in this experiment showed delayed S and G2/M phase progression and accumulated CyclinB1 in HeLa cells (**Fig. S4**). We noted that both etoposide and 5FU augmented the cell death effect of Cdc7 inhibition in p53-positive HCT116 but not in p53-negative cells (**Fig. 9**). It is speculated that cell death during S phase in Cdc7-inhibited p53-positive HCT116 is further stimulated by the inhibition of DNA chain elongation through etoposide or 5FU. Meanwhile, in p53-negative HCT116 cells, cell death, induced mostly by aberrant M phase progression from G2-arrest, is not affected significantly by the added S phase inhibitions. Similar effect of etoposide on cancer cell death induced by Cdc7 depletion was previously reported [41]. These results suggest potentially effective cancer therapy strategies depending on the genotype of tumors. In p53-positive cancer cells, a combination of inhibitors of DNA replication initiation and genotoxic agents interfering the DNA chain elongation process may be an effective measure for cell death induction, whereas combination of Cdc7 inhibition with genotoxic agents targeting G2-M phase progression could be an effective measure in p53-negative cancer cells. The latter possibility is now being tested.

**Figure 10:**
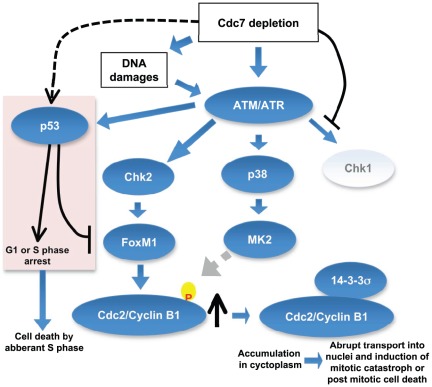
Proposed pathways of cell death induced in cancer cells by inhibition of Cdc7 kinase. Inhibition of initiation of DNA replication by suppression of Cdc7 kinase leads to activation of ATM/ATR, which may result in the activation of three checkpoint kinases, Chk1, MK2, and Chk2. Since Cdc7 is actively required for activation of Chk1 [19], [46], Chk1 is not activated under this condition. Activated MK2 may phosphorylate Cdc2/Cyclin B1, which in turn may be recognized and bound by 14-3-3σ protein and is sequestered in cytoplasm. Cdc7 depletion can induce DNA damages in cancer cells [19] and activated Chk2 would stabilize the FoxM1 transcription factor, which would induce the expression of CyclinB1 [47]. The accumulated CyclinB1 protein is abruptly transported into nuclei and mitotic catastrophe or post-mitotic cell death is induced. In p53-positive cancer cells, p53, activated through ATM/ATR, would induce G1 delay as well as S phase delay possibly through induction of p21. p53 inhibits transcription of FoxM1 [37], [38], thus preventing the induction of Cyclin B1. However, aberrant S phase progression in the absence of Cdc7 would induce cell death in p53-positive cancer cells.

In summary, we show that different cell death pathways are induced in cancer cells by inhibition of Cdc7 kinase, depending on the p53 status (**Fig. 10**). Cdc7 depletion would induce “defective initiation” which may send checkpoint signals directly to ATM/ATR or through DNA damages caused by aberrant initiation of DNA replication in the absence of Cdc7. In the absence of p53, aberrant S phase may proceed to completion but the activated checkpoint could induce G2 elongation through MK2, eventually leading to post-mitotic cell death. In the presence of p53, the initiation defect caused by Cdc7 inhibition may predominantly cause transient G1 or S phase arrest. Aberrant progression into S phase and generation of pathological stalled fork structures under these conditions may lead to collapsed replication forks and generate lethal DNA damages, leading to cell death in S phase. A p53-induced pro-apoptotic factor may also contribute to cell death. In normal cells with wild-type p53 and all other checkpoint machinery functioning, a defect in initiation would be effectively detected and stalled before entering abortive S phase, thus permitting the cells to escape from cell death [16], [42].

## Materials and Methods

### Cell lines and the cells expressing fluorescence-tagged proteins

All cells including HeLa, U2OS, HCT116 (p53-positive), NHDF and 293T cells were obtained from ATCC, and were maintained as described previously [5], [15], [19]. Lentiviruses for expressing fluorescence-tagged proteins were generated as described previously [18]. mKO2-CyclinB1 and mKO2-AuroraA expressing plasmids were constructed by replacing the Cdt1 part of the mKO2-Cdt1 vector with the full-length CyclinB1 and AuroraA, respectively. p53-negative HCT116 cells were obtained from Dr. B. Vogelstein.

### Preparation of extracts, western blotting and immunoprecipitation

Cell extract preparation, western blotting and immunoprecipitation were conducted as described previously [5], [19].

### siRNA, antibodies and inhibitors

siRNAs and antibodies used are described in supplementary information (**Table S1**). The Cdk9/Cdc7 inhibitor was from Calbiochem.

### Transfection

Plasmid DNA transfection into 293T cells was conducted by using Lipofectamine2000 reagent or PEI solution [43], [44] to prepare lentiviral solutions. Plasmid DNA transfection into HeLa was conducted by using FuGENE HD (Roche). Oligofectamine transfection reagent (Invitrogen) was used for transfection of siRNA into HeLa, U2OS and NHDF cells, and X-tremeGENE siRNA transfection Reagent (Roche) was used for siRNA transfection into HCT116 cells.

### Time lapse analyses of fluorescent cells

For live cell imaging, cells were plated in a glass-bottomed dish, μ-Dish (Ibidi), transfected with siRNAs and observed under LCV100 microscopy (Olympus) equipped with an objective lens (Olympus, UAPO 403/340 N.A. = 0.90). For fluorescence imaging, the halogen lamp was used with two filter cubes, one for observing mKO2 fluorescence (with excitation [BP520-540HQ] and emission [BP555-600HQ] filters), and the other for observing mAG fluorescence (with excitation [470DF35] and emission [510WB40] filters). For DIC imaging, the red LED was used with a filter cube containing an analyzer. Image acquisition and analyses were performed by using MetaMorph software version 7.1.3 and 7.5.1 (Universal Imaging, Media, PA), respectively. Prism software (GraphPad Software Co. Ltd.) was used for analyses of indicated signals from acquired movies and for two-tailed unpaired t-test.

### Immunostaining

For immunostaining, cells were fixed with 4% paraformaldehyde for 10 min at room temperature, washed with PBS, permealysed with 0.1% Triton X-100, and stained by anti-CyclinB1 antibody overnight at 4°C. After washing, the cells were stained with FITC-conjugated anti-mouse IgG for 30 min at room temperature, followed by Hoechst staining. Antibodies were diluted with dilution buffer (2 mg/ml BSA, 0.2% Tween20 and 10% glycerol). Stained cells were observed under All-in-One microscopy (Keyence) or FSX100 microscopy (Olympus).

### *In vitro* kinase assays of Cdc2-CyclinB1

Cdc2/CyclinB1 kinase activity was measured by incubating the anti-CyclinB1 antibody immunoprecipitate with Histone H1 as a substrate, as described previously [45].

### Analyses of proteins on phosgel

10% SDS-PAGE gel containing 25 µM phos-tag AAL-107 (NARD Institute Ltd.) and 50 µM MnCl_2_ was used to separate phosphorylated proteins according to the manufacture's instruction.

## Supporting Information

Figure S1 Cdc7 depletion in cancer and normal cells. (**A**) FACS analyses of HeLa or U2OS cells (10,000 cells for each) treated with control (green) or Cdc7-D (red) siRNA for times indicated. Sub-G1 population increased after Cdc7 depletion in both cell lines. (**B**) FACS analyses of NHDF cells (10,000 cells for each) treated with Cdc7-D or control siRNA for 48 hrs. BrdU was incorporated into cells for 30 min and the harvested cells were stained with anti-BrdU antibody and PI as described in ”Materials and Methods”. (**C**) Western blot analyses of the whole cell extracts of NHDF cells treated with control or Cdc7-D siRNA for 48 hrs. In Cdc7-depleted NHDF cells, CyclinB1 protein level decreased with concomitant decrease of mitotic cells (indicated by the decreased phosphorylated serine 10 signal of Histone H3). (**D**) NHDF cells expressing Fucci were treated with Cdc7-D (lower) or control siRNA (upper) and time lapse images were recorded. Images taken from the time lapse data at the times indicated are presented. Red cells accumulate in Cdc7 siRNA-treated NHDF cells, indicating the arrest in G1 phase.
(EPS)

Figure S2 Accumulation of CyclinB1 in the cytoplasm and cell death are observed with other siRNA in HeLa cells. (**A**) The time (hr) between the first appearance of cytosolic mKO2-CyclinB1 signal and its translocation into nucleus was measured in the time lapse images of HeLa/mKO2-CyclinB1 cells treated with control or various Cdc7 siRNAs (Cdc7-D, Cdc7-A and Cdc7-new1). Cytoplasmic accumulation of Cdc7 is observed with Cdc7-A and Cdc7-new1 siRNAs as well. (**B**) FACS analyses of HeLa cells (10,000 cells for each) treated with control (green) or Cdc7-A (red) siRNA for times indicated. Sub-G1 population increased after Cdc7 depletion.
(EPS)

Figure S3 Cdc7 depletion in HeLa cells expressing mKO2-AuroraA. (**A**) HeLa cells stably expressing mKO2-AuroraA were established and treated with control (left) or Cdc7-D (right) siRNA. Time lapse images were recorded by Olympus LCV100. Images taken from the time lapse data at the times indicated are shown. Red signals show mKO2-AuroraA. Cdc7 depletion leads to increased duration of red signals and subsequent cell death, suggesting that cells are arrested in G2 phase before undergoing cell death. Bar: 20 µm. (**B**) HeLa cells expressing mKO2-AuroraA were cultured on glass-bottomed dishes and were labeled with EdU for 10 min. Cells stained with Crick IT EdU detection kit (Invitrogen) were observed by FSX100 microscopy (Olympus). Green, EdU (DNA synthesis); red, mKO2-AuroraA. mKO2-positive cells and EdU positive cells do not overlap. Bar: 153 µm. (**C**) The time (hr) between the first appearance of nuclear mKO2-AuroraA signals and the cells' transition into prophase (round cells) was measured from the time lapse data and is presented for control or Cdc7 siRNA (Cdc7-D, -A and -new1)-treated HeLa cells (**movies S5 and S6**). The P-values of the two-tailed unpaired t-test were calculated by Prism software.
(EPS)

Figure S4 Cdc7 inhibitor delayed cell cycle progression with accumulated CyclinB1 protein in HeLa. (A) HeLa cells were grown in the presence of 2.5 mM thymidine for 15–16 hrs, followed by successive growth without thymidine for 9 hrs and with thymidine for 15–16 hrs. The G1/S-arrested cells were released into cell cycle for indicated times in the presence of 1 µM Cdc7 inhibitor or DMSO and FACS and Western analyses were conducted. The Cdc7 inhibitor delayed S, G2/M phase progression and accumulated CyclinB1. (**B**) HeLa (left) and U2OS (right) cells were treated with 1 µM Cdc7 inhibitor for 24 hrs and harvested. FACS and Western analyses were conducted. CyclinB1 accumulation was observed only in HeLa cells.
(EPS)

Table S1 siRNAs and antibodies used in this study. (EPS)

Movie S1 HeLa cells expressing Fucci (mKO2-hCdt1 (red) for G1 and mAG-hGeminin (green) for S/G2/M) were treated with control siRNA and time lapse image was recorded by LCV100 Microscopy (Olympus). Both videos start at 24 hrs after siRNA transfection. Total imaging time was 72 hr. Images were acquired every 60 min. Playback speed is 21600× real time.
(AVI)

Movie S2 HeLa cells expressing Fucci (mKO2-hCdt1 (red) for G1 and mAG-hGeminin (green) for S/G2/M) were treated with Cdc7-D siRNA and time lapse image was recorded by LCV100 Microscopy (Olympus). Both videos start at 24 hrs after siRNA transfection. Total imaging time was 72 hr. Images were acquired every 60 min. Playback speed is 21600× real time.
(AVI)

Movie S3 HeLa cells expressing mKO2-CyclinB1 (red) were treated with control and time lapse image was recorded by LCV100 Microscopy (Olympus). Both videos start at 24 hrs after siRNA transfection. Total imaging time was 42 hr. Images were acquired every 40 min. Playback speed is 13745× real time.
(AVI)

Movie S4 HeLa cells expressing mKO2-CyclinB1 (red) were treated with Cdc7-D siRNA and time lapse image was recorded by LCV100 Microscopy (Olympus). Both videos start at 24 hrs after siRNA transfection. Total imaging time was 42 hr. Images were acquired every 40 min. Playback speed is 13745× real time.
(AVI)

Movie S5 HeLa cells expressing mKO2-AuroraA (red) were treated with control siRNA and time lapse image was recorded by LCV100 Microscopy (Olympus). Both videos start at 24 hrs after siRNA transfection. Total imaging time was 60 hr. Images were acquired every 40 min. Playback speed is 14400× real time.
(AVI)

Movie S6 HeLa cells expressing mKO2-AuroraA (red) were treated with Cdc7-D siRNA and time lapse image was recorded by LCV100 Microscopy (Olympus). Both videos start at 24 hrs after siRNA transfection. Total imaging time was 60 hr. Images were acquired every 40 min. Playback speed is 14400× real time.
(AVI)
